# Validation of the Clinical Assessment Scale in Autoimmune Encephalitis in Chinese Patients

**DOI:** 10.3389/fimmu.2021.796965

**Published:** 2021-12-17

**Authors:** Yingchi Zhang, Ewen Tu, Chenxiao Yao, Jia Liu, Qiang Lei, Wei Lu

**Affiliations:** ^1^ Department of Neurology, The Second Xiangya Hospital, Central South University, Changsha, China; ^2^ Department of Neurology, The Second Hospital of Hunan Province, Hunan University of Chinese Medicine, Changsha, China

**Keywords:** autoimmune encephalitis, CASE scores, mRS scores, validation, prognosis

## Abstract

**Background and Objectives:**

The Clinical Assessment Scale in Autoimmune Encephalitis (CASE) is a scale for assessing severity in autoimmune encephalitis. We aimed to validate the CASE score in a Chinese population and evaluate its clinical significance.

**Methods:**

Patients diagnosed with autoimmune encephalitis were recruited between June 2014 and May 2019 from two hospitals. CASE and modified Rankin Scale (mRS) scores were obtained. Data regarding clinical features, treatment, and available information were gathered from the hospital information system.

**Results:**

Of the 176 patients with autoimmune encephalitis, 11 died and 14 had tumors. Ten patients received second-line treatment. The CASE scores of patients receiving second-line treatment were significantly higher (median CASE: 15) than in those receiving first-line treatment (median CASE: 8) (p<0.001). Twenty-two patients had poor functional status (mRS>2). Areas under the curve of CASE on whether functional status was poor at 1 year were 0.89 (p<0.001). Sixty patients were admitted to the intensive care unit (ICU), and the CASE scores were positively correlated with days in the ICU (r=0.58, p<0.001). There was no statistically significant association between the CASE scores and relapse (p=0.39>0.05). Additionally, the CASE scores were positively associated with the mRS scores (r=0.85 p<0.001).

**Conclusions:**

The CASE score is suitable for the comprehensive assessment of Chinese patients with autoimmune encephalitis, which may help clinicians to select the appropriate intervention and estimate the disease severity and prognosis.

## Introduction

Autoimmune encephalitis (AE) is an autoimmune-mediated neurological disease characterised by an acute or subacute onset of psychiatric or neurological symptoms (1–4). Its main clinical symptoms include abnormal psychiatric behaviour, cognitive impairment, memory loss, seizures, speech disorders, movement disorders, involuntary movements, decreased level of consciousness, and autonomic dysfunction (5–7). Currently, AE is responsible for 20% of all types of encephalitis (8), of which anti-N-methyl-d-aspartate receptor (NMDAR) encephalitis is the most common, accounting for approximately 80% of AEs, followed by leucine-rich glioma inactivated 1(LGl1) antibody, anti-gamma-aminobutyric acid-B receptor (GABABR) antibody, anti-alpha-amino-3-hydroxy-5-methyl-4-isoxazolepropionic acid receptor (AMPAR) antibody, and anti-dipeptidyl-peptidase-like protein 6 (DPPX) antibody encephalitis (9–12). Recent advancements in AE treatment include the establishment of immunotherapy treatment, such as first-line (steroids, intravenous immunoglobulin [IVIG], and plasma exchange) and second-line (rituximab and cyclophosphamide) treatment (13). AE often has a good clinical outcome if patients are promptly diagnosed and treated (14). Different immunotherapy regimens are currently recommended depending on the severity of the disease (15). Therefore, it is important to accurately assess the severity of the disease in patients with AE (16).

Currently, almost all clinical studies related to AE use the modified Rankin Scale (mRS) to assess the severity and prognosis of AE (17, 18). However, the mRS is designed to assess disability after stroke (19), and there are significant limitations in the assessment of AE. For example, the mRS is weighted by motor ability. However, patients with AE have a wide variety of clinical symptoms in addition to compromised motor function. Early identification of patients and accurate assessment of the severity of their disease are considered major problems in clinical practice.

The Clinical Assessment Scale in Autoimmune Encephalitis (CASE) score (20) is the first score designed specifically to assess the severity of AE. It is a general scoring system ranging from 0 to 27 and can be used as an alternative tool to assess the severity of patients with AE more accurately. Thus, this study aimed to verify the validity of CASE scores.

## Methods

### Data Source

Patients with AE were studied consecutively in two clinical centres in Hunan, China. Based on the definition of AE in the latest consensus declaration (5), we included patients who met the criteria for AE. The inclusion criteria were as follows (1): patients aged >6 years; (2) patients with acute AE onset with one or more of the following clinical symptoms: psychosis, impaired memory, impairment of speech, seizures, dyskinesias, loss of consciousness, disordered autonomic function, and central hyperventilation; (3) diagnosed with antibody-positive AE; and (4) patients with at least one systemic screening for tumors.

The exclusion criteria were as follows: (1) patients with confirmed diagnosis of infectious encephalitis (with evidence of laboratory tests) caused by viruses, bacteria, *Mycobacterium tuberculosis*, parasites or fungi, and cryptococci; (2) patients diagnosed with toxic/metabolic encephalopathy, brain tumors, vitamin deficiency or alcohol-associated encephalopathy, and seizures and/or other neurological disorders before the development of AE; (3) patients with encephalitis of unknown aetiology; (4) patients who refused to participate in the study; and (5) patients with insufficient key clinical data.

Antibodies testing were done through indirect immunofluorescence testing (IIFT) or cell based assays in the Guangzhou King Med Center for Clinical Laboratory.All samples are tested at the admission and patient data are available in the hospital information system.

This study was approved by the Second Xiangya Hospital ethics review board. Written informed consent was obtained from the patients.

### Study Populations

**Figure 1:**
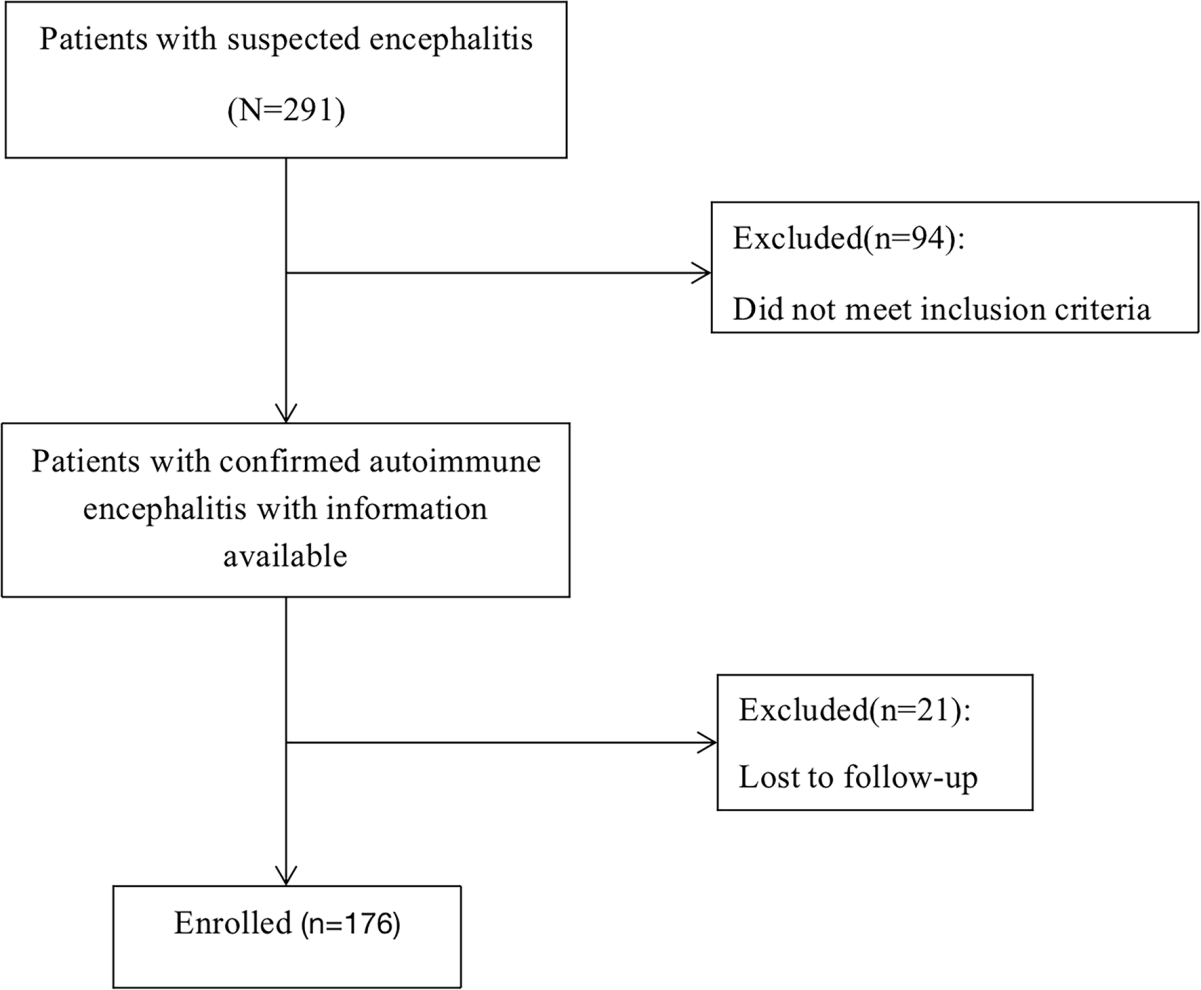
Flow chart of study design.

We enrolled individuals with AE who had available data between 1 June 2014 and 1 May 2019. All participants signed a written informed consent before the start of the study. A total of 291 patients were enrolled, 94 of whom were excluded due to the diagnosis of other diseases. 21 (10.66%) were lost to follow-up(**Figure 1**), and they are patients who lack at least one of the 3-6-12-24 months visits.

### Data Collection

This study collected patients’ baseline demographics, time to symptom onset, time to symptom improvement, clinical characteristics, treatment regimens, the length of stay (LOS) in the hospital, and details of the nine variables involved in the CASE scores at admission.

All patients with AE were scored on admission (before treatment).After discharge, clinical information was collected face-to-face, by telephone or Wechat by two neurologists who are experts in autoimmune encephalitis, and CASE scores were assessed at 3, 6, 12, and 24 months after discharge. If there was any disagreement, a third senior neurologist was called upon to reach a consensus. For patients admitted before 2019, the scale was assessed retrospectively by a neurologist who was unaware of the study through detailed medical records described by the neurologist and nurse at the time of admission. This is a study conducted at a neuroimmune center in Hunan and the investigators are also neuroimmunologists.

### Clinical Assessment Scale in Autoimmune Encephalitis (CASE) Score

The CASE was developed from a study conducted by Lim et al., which describes a new scale to assess the severity of AE and the effectiveness of treatment(20). The scale includes nine items(**Table 1**): seizures, memory dysfunction, psychiatric symptoms, consciousness, language problems, dyskinesia/dystonia, gait instability and ataxia, brainstem dysfunction, and muscle weakness. It has a subitem score of 0–3 for each key item, with a sum of nine key items and a maximum score of 27.

**Table 1:** Clinical assessment scale for autoimmune encephalitis (CASE).

Key symptom	Scale	Score
Seizure	None	0
	Controlled seizures	1
	Intractable seizures*	2
	Status epilepticus	3
Memory dysfunction	None	0
	Mild (does not affect daily activities)	1
	Moderate (interferes with daily activities)	2
	Severe (no recent memory or unable to communicate)	3
Psychiatric symptoms	None	0
	Mild (no need for medical intervention because it does not affect daily activities)	1
	Moderate (need for medical intervention because it interferes with daily activities)	2
	Severe (needs continuous care or admission because of psychiatric symptom) or unable to check	3
Consciousness	Alert (opens eyes spontaneously)	0
	Drowsy (opens eyes to voice)	1
	Stupor (opens eyes to pain)	2
	Comatose (does not open eyes)	3
Language problem	None	0
	Mild (slow but able to express sentences)	1
	Moderate (unable to express full sentences)	2
	Severe (unable to communicate)	3
Dyskinesia/dystonia	None	0
	Mild dyskinesia (does not affect daily activities)	1
	Moderate dyskinesia (interferes with daily activities)	2
	Severe dyskinesia causing secondary medical problems	3
Gait instability and ataxia	Normal	0
	Mild, able to walk unassisted	1
	Moderate, assisted walking	2
	Severe, unable to walk	3
Brainstem dysfunction (number of symptoms)	None	0
	Gaze paresis	1
	Tube feeding	2
	Ventilator care due to central hypoventilation	3
Weakness (the mean motor power of all limbs, rounded off)	Normal (Grade V)	0
	Mild (Grade IV)	1
	Moderate (Grade III)	2
	Severe (≤ Grade II)	3

The copyright for the CASE score is owned by Seoul National University Hospital. For inquiries, please contact ip@snuh.org or staelee@snu.ac.kr.

### Evaluation of Prognosis and Operational Definitions

Follow-up information was assessed by clinicians after disease diagnosis, and was objectively assessed by experienced neurologists. Clinical relapse was characterised as a new onset or further deterioration of pre-existing conditions that occurred at least 2 months after the initial improved or stabilized condition (5, 21).

Good and poor functional statuses were determined as an mRS score ≤2 and an mRS score >2, respectively, after 12 months of follow-up. Early and timely treatment was considered as starting immunotherapy within 4 weeks of onset. We also defined clinical outcomes according to the CASE score as excellent (0–4), moderate (5–9), or poor (10–27) (14).

### Statistical Analyses

Categorical variables are shown as frequencies. Parametric continuous variables are described as the mean; nonparametric variables are presented as the median. χ^2^ statistics or Fisher’s exact tests were used for baseline variables. The Mann-Whitney U test was used for continuous variables for two-sample variables. The Kruskal-Wallis test was used for continuous variables of multisamples. For the multivariate analysis of LOS, four variables were pre-selected to investigate the relationship with LOS (age, mRS at admission, the length of ICU stay, and CASE scores at admission). The power to discriminate CASE scores was assessed by the area under the receiver operating characteristic curves (AUCs) and 95% CI. An AUC of 1.0 represents a perfect prediction, and that of 0.5 represents a prediction that was considered to not be any better than a casual prediction. A p-value <0.05 was considered statistically significant. All analyses were performed using the Statistical Package for the Social Sciences (version 25; International Business Machines Corporation, Armonk, NY).

## Results

### Patient Characteristics

This study comprised 176 patients from two hospitals with a median age of 28 (6–86) years, and 83 females (46.63%) were included in this study. In the validation cohort, 140 (79.55%) patients were diagnosed with anti-NMDAR encephalitis, 21 (11.93%) with GABABR antibody encephalitis, 12 (6.82%) with LGl1 antibody encephalitis, two (1.14%) with DPPX antibody encephalitis, and one (0.57%) with AMPAR antibody encephalitis. The median mRS score at admission for the patients in the validation cohort was 3.0 (mRS 1: n=3, 2: n=63, 3: n=69, 4: n=12, 5: n=29). The median time to diagnosis was 4 days(range:1-7), The median time to treatment was 5 days(range:1-8).

The baseline features of the patients are summarised in **Tables 2** and **3**. The most common clinical presentation was psychiatric symptoms (89.90%). Fourteen patients had an underlying tumor, 6(42.86%) with ovarian teratoma, 3(21.43%) with lung cancer, 3(21.43%) with bladder cancer,and 2(14.28%) with thyroid Cancer. During the first year, 11 (6.25%) patients died, whereas 27 (15.34%) patients experienced relapse. Moreover, 156 (88.64%) patients had a good clinical outcome (mRS score ≤2).

**Table 2:** Characteristics of patients with autoimmune encephalitis.

Clinical characteristics	All (n = 176)	Age<18 y (n = 39)	Age≥18 y (n = 137)	p value
Median age, range (y)	28 (6-86)	15 (6–17)	31 (18–86)	<0.0001
Female	83 (46.63)	24 (61.54)	59 (43.07)	0.04
Seizures	114 (64.77)	30 (76.92)	84 (61.31)	0.72
Memory dysfunction	131 (74.43)	27 (69.23)	104 (75.91)	0.399
Psychosis	158 (89.77)	37 (94.87)	121 (88.32)	0.373
Decreased level of consciousness	110 (62.50)	27 (69.23)	83 (60.58)	0.325
Language problem	118 (67.05)	29 (74.36)	89 (64.96)	0.271
Dyskinesia	39 (22.16)	9 (23.08)	30 (21.90)	0.876
Gait instability and ataxia	22 (12.50)	6 (15.38)	16 (11.68)	0.732
Brainstem dysfunction	68 (38.64)	15 (38.46)	53 (38.69)	0.98
Admitted to the ICU	60 (34.09)	16 (41.03)	44 (32.12)	0.30

**Table 3:** Frequency of distributions of symptoms between ANRE and Non-ANRE.

Clinical characteristics	All(n = 176)	Non-ANRE(n = 36)	ANRE(n = 140)	p value
Seizures	114(64.77)	30(83.33)	84(60.00)	0.009
Memory dysfunction	131(74.43)	22(61.11)	109(77.86)	0.04
Psychosis	158(89.77)	27(75.00)	131(93.57)	0.003
Decreased level of consciousness	110(62.50)	21(58.33)	89(63.57)	0.563
Language problem	118(67.05)	22(61.11)	96(68.57)	0.396
Dyskinesia	39(22.16)	7(19.44)	32(22.86)	0.660
Gait instability and ataxia	22(12.50)	8(22.22)	14(10.00)	0.090
Brainstem dysfunction	68(38.64)	16(44.44)	52(37.14)	0.422
Muscle weakness	46(26.14)	14(38.89)	32(22.86)	0.051

ANRE:anti-NMDAR encephalitis

### CASE Score and Treatment

**Figure 2:**
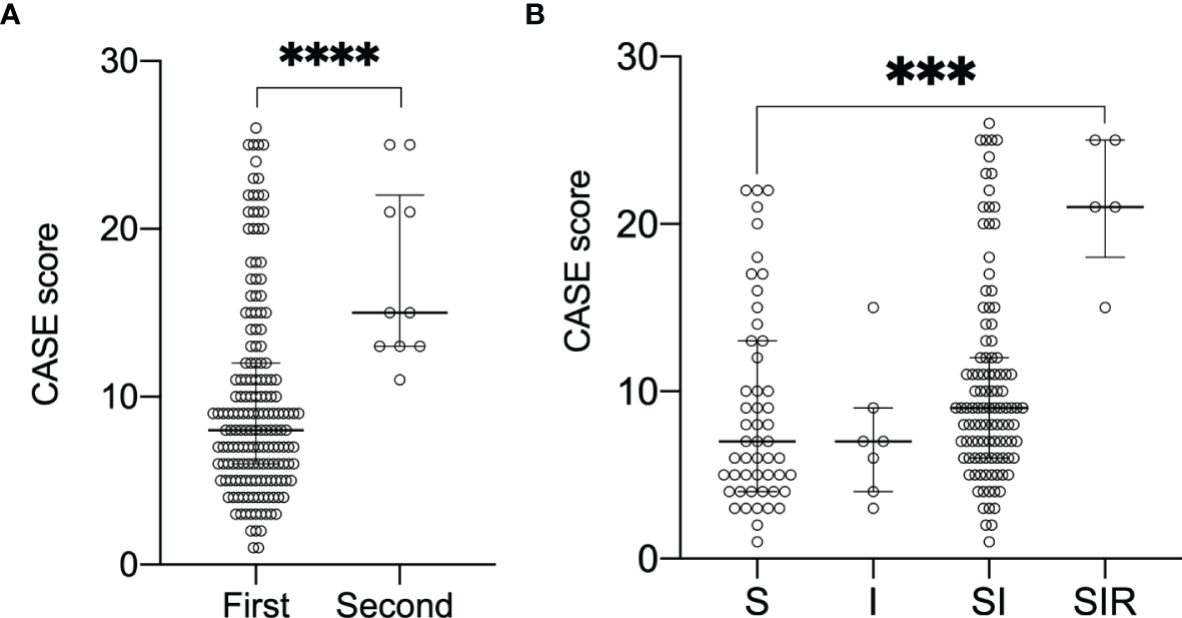
Change of CASE scores according to the different treatment regimens. Data are reported as median (interquartile range, IQR). **(A)** demonstrates CASE score with first-/second-line treatment(****P<0.0001 Mann-Whitney test). **(B)** demonstrates CASE score and S/I/SI/SIR(***P<0.001 Kruskal-Wall test).Abbreviations:S steroid; I intravenous immunoglobulin; SI steroid and IVIG;SIR steroid, IVIG and rituximab; CASE Clinical Assessment Scale in Autoimmune Encephalitis.

All patients received first-line therapy (glucocorticoids, IVIG alone or in combination), and only 10 (5.68%) patients received second-line immunotherapy (rituximab, cyclophosphamide alone or in combination). The median CASE score of patients receiving second-line treatment was 15 (**Figure 2A**), whereas the median CASE score of patients receiving first-line treatment was only 8 (p<0.001 Mann–Whitney U test). Thus, patients receiving second-line treatment had higher CASE scores than patients receiving first-line treatment. More specifically, the SIR (steroid, IVIG, and rituximab) group had higher CASE scores compared to the S/I/SI group (steroid only/IVIG only/steroid and IVIG) (**Figure 2B**, p<0.001, Kruskal–Wallis test).

### CASE Score and Risk of Poor Prognosis

**Figure 3:**
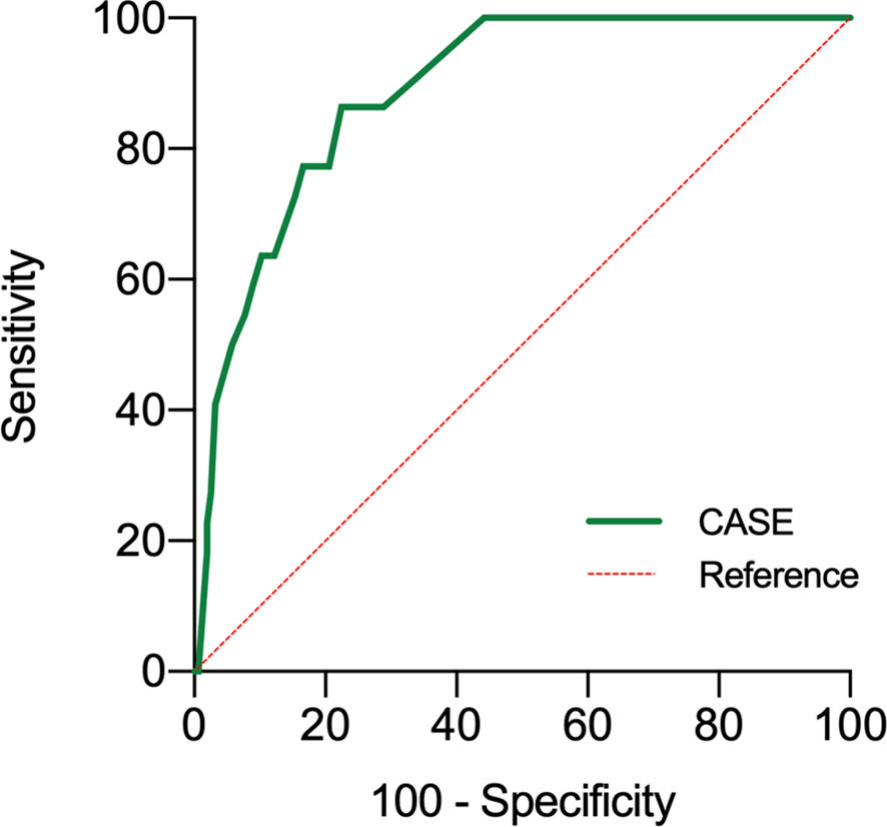
Receiver operating characteristic curve for the prediction of 1-year prognosis of the CASE. Poor functional statuses were determined as an mRS score >2 at 1 year. CASE Clinical Assessment Scale in Autoimmune Encephalitis, at admission before treatment.

**Figure 4:**
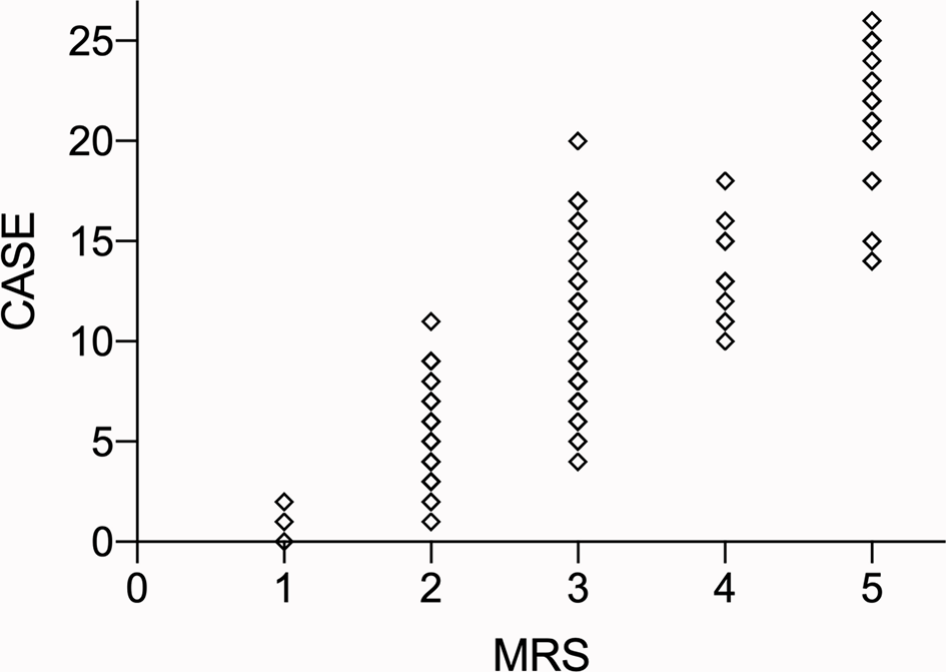
The total CASE score according to the mRS. Abbreviations: CASE: Clinical Assessment Scale in Autoimmune Encephalitis; NEOS: anti-NMDAR Encephalitis One-Year Functional Statu; mRS: modified Rankin Scale.

The area under the curve (AUCs) of the CASE scores are shown in **Figure 3**. The CASE score proved to be a significant predictor of poor functional status in AE (AUC: 0.89, 95% confidence interval [CI]: 0.83–0.95, p<0.05). Additionally, the CASE scores were positively correlated with the mRS score (**Figure 4**) (r: 0.85, 95% CI: 0.80–0.89).

### CASE Score and Relapse

Twenty-seven (15.34%) patients experienced clinical relapses, 16 of whom (59.30%) experienced multiple relapses. However, the mRS scores, CASE scores, and relapses did not have a statistically significant association (p>0.05).

### CASE Score and Length of Stay in the Hospital

Of the 83 patients having an LOS ≥30.0 days, 67 were in the NMDAR group, 8 were in the GABABR group, 8 were in the LGl1/DPPX group. The median LOS of patients was 28 (range 3–154) days.The factors influencing prolonged LOS included high CASE scores (p<0.05), and long length of ICU stay (p<0.001 multiple linear regression). There was no significant difference in age (p=0.64) and mRS (p=0.42).

**Figure 5:**
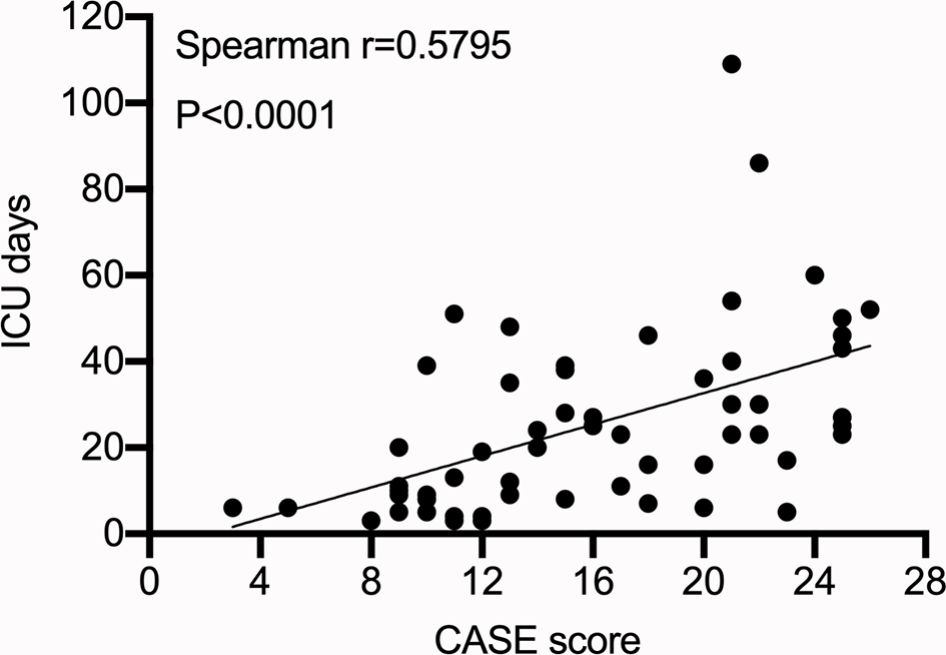
CASE score at admission and intensive care unit stay.

Moreover, 60 (34.09%) patients were admitted to the intensive care unit (ICU), of which 80% of the patients were admitted to the ICU for persistent epilepsy (CASE score: seizure=3). The CASE score was positively correlated with the length of ICU stay (**Figure 5**) (p<0.05 Spearman’s correlation).

## Discussion

To the best of our knowledge, this is the largest autoimmune encephalitis cohort to validate the CASE scores in China. The main findings of the study are as follows: (1) The CASE score can efficiently predict the probability of poor functional status at 1 year after discharge in Chinese patients with AE.(2) The higher the CASE score in baseline, the longer the hospital stay and ICU stay, which may increase the patients’ financial burden.(3)CASE scores were positively correlated with the mRS score.

CASE scores were also validated by Cai et al. (2021) (22), who suggested that there was a good correlation between CASE and mRS scores at the time of admission (r=0.80, p<0.001); this is consistent with our findings (r=0.85 p<0.05). We found that the most common clinical presentation in our study was psychiatric symptoms. Cai et al. also reported that the largest changes with respect to non-motor symptoms corresponded to psychosis. This further demonstrates that psychiatric abnormalities are important clinical symptoms of autoimmune encephalitis in China.

Our study revealed a lower prevalence of AE in females (46.63%) than in males, as well as a lower tumor rate (7.95%) in China than in the United States (11, 23–27). This is similar to other AE-related reports from China. For example, Yan Zhang et al. (2018) showed that among 111 patients with NMDAR-antibody encephalitis including 59 males (53.2%) and 52 females (46.8%), nine (9/111 8.1%) patients had combined ovarian teratomas (28).In a retrospective study by Wei Shan et al., the ratio of males to females with autoimmune encephalitis was 1.2:1, and tumors accounted for only 4.4% of the patients (29).However, according to some studies conducted in the United States and Europe, >80% of patients with AE were females, and 20–59% had tumors (11, 23–27).

Moreover, only 33.71% of Chinese patients were sent to the ICU (6, 30), whereas other study cohorts had ICU admission rates as high as 50–77%. The recurrence rate in our study was 15.17%, whereas in some studies, it was as high as 36.4% (31, 32). Some experts believe that race-specific factors, human leukocyte antigens, or other genetic factors may play an important role in the development of AE.

It is difficult to determine when and whether to initiate second-line therapy for AE patients. The CASE score may be an index for initiating second-line treatment. For example, our data showed that patients receiving second-line treatment had higher CASE scores than patients receiving first-line treatment, the median CASE score for patients receiving second-line treatment was 15, which provides insights and data to support clinicians in optimising treatment options.

However, only 5.68% of patients in our cohort received second-line immunotherapy regimens; therefore, the statistical data analysis regarding first-line versus second-line treatment does not seem to be significantly reliable, which is significantly different from the US study (17.0–27.0% of patients) (11, 25). This phenomenon observed in our cohort could be due to the patients’ financial reasons and the physicians’ concerns regarding side effects.

Additionally, since the CASE score mainly assesses the patient’s clinical symptoms, it can be continuously monitored to assess the effectiveness of treatment or to record the course of the patient’s score during follow-up. The NEOS score, a new score (**Table 4**) generated in 2019 by Dalmau, which predicts 1-year functional status in patients with anti-NMDAR encephalitis and is also validated by Chinese studies (33, 34), is difficult to assess continuously for every relapsed patient because not all patients with AE are reviewed regularly with imaging and cerebrospinal fluid tests.

**Table 4:** Anti-NMDAR Encephalitis One-Year Functional Status (NEOS) score.

Items	Score
ICU admission	1
the absence of treatment for more than 4 weeks	1
improvement delay of more than 4 weeks after starting treatment	1
abnormal MRI	1
CSF white blood cell (WBC) count of more than 20 cells/μL	1

However, in some patients with status epilepticus or mechanical ventilation, clinicians often use drugs to sedate the patients, so the patient is unable to cooperate with clinicians, which made new challenges in obtaining the CASE scores.When patients are sedated, they are unable to speak. Thus, we are unable to accurately assess the patient’s level of consciousness, language and memory, which are important items of CASE score.

This study revealed several features that were inconsistent with the original CASE study. First, during our application of the CASE rating scale, we found that the majority of patients with AE in China (89.9%) had psychosis. According to some studies in Western countries, approximately 58% of patients had psychosis, which is lower than that in China. Subsequently, in clinical practice, it would be particularly difficult to evaluate the level of consciousness in patients with psychosis; thus, our institution mainly evaluates the content of consciousness (confusion, delirium) in patients with AE. Therefore, in the future, strategies on how to score the consciousness in patients with AE may need further improvement.

The CASE has strengths over the modified Rankin scale in better evaluating non-motor symptoms, such as psychiatric symptoms, language and memory dysfunctions. In other words, CASE compensates for the limitations of the mRS in terms of non-motor symptoms. However, there are some problems when CASE is applied in clinical practice. For example, memory and language problems are often difficult to assess when the patient is drowsy or stupor, in which the clinicians may prefer to use mRS score as a coarse and simplistic assessment for AE patients, similar to that for patients with stroke in whom both the National Institutes of Health Stroke Scale (NIHSS) and Glasgow Coma Scale(GCS) scores can be obtained. This is because in patients experiencing loss of consciousness, NIHSS scores seem difficult to obtain to accurately assess the patient’s condition, and the GCS scores can compensate for the lack of NIHSS scores in this regard. Thus, it may be possible to combine the CASE and mRS scores to assess the severity of AE.

Our study has a few limitations. First, our study only included two large hospitals, with better access to medical facilities and specialists than small hospitals in urban areas; therefore, selection bias may have been present. Second, we did not follow up all patients for a longer duration, and in the future, we may be able to use the CASE score to conduct a prospective study of all patients with AE. Third, since the number of people receiving second-line therapy in China was significantly small, the relevant results derived may need to be interpreted with caution. More importantly, for paediatric patients, second-line immunotherapy may be limited for reasons of safety when first-line therapy is not successful. Finally, our study lacked the usage of biomarkers related to treatment response, which may reduce the ability of the score to predict final clinical outcomes.

For example, neurofilament light chains (Nf-L) and heavy chains (Nf-H) were used, as they are efficient biomakers of nerve damage (35). A study by Jiayu Li et al. (2019) showed that CSF Nf-L was positively associated with mRS scores in patients with anti-NMDAR encephalitis. They found that CSF Nf-L and Nf-H levels decreased after treatment (35). Moreover, Nf-L levels were positively correlated with mRS scores at the 3-month follow up (35). Therefore, we are likely to investigate whether Nf-L is associated with CASE in the future.

In conclusion, the CASE score was shown to be suitable for assessing Chinese patients with AE, which can help clinicians evaluate the severity of the disease and the potential prognosis. A comprehensive assessment of patients using the newly developed CASE scales should be attempted in the future.

## Data Availability Statement

The raw data supporting the conclusions of this article will be made available by the authors, without undue reservation.

## Ethics Statement

The studies involving human participants were reviewed and approved by Second Xiangya Hospital ethics review board. The patients/participants provided their written informed consent to participate in this study.

## Author Contributions

Study designed by WL and QL. Data collected by ET and CY. YZ and JL drafted the manuscript and figures. All authors participated in the revision of the manuscript. All authors contributed to the article and approved the submitted version.

## Funding

This work was supported by the Natural Science Foundation of Hunan Province (Grant No. 2018JJ6014).

## Conflict of Interest

The authors declare that the research was conducted in the absence of any commercial or financial relationships that could be construed as a potential conflict of interest.

## Publisher’s Note

All claims expressed in this article are solely those of the authors and do not necessarily represent those of their affiliated organizations, or those of the publisher, the editors and the reviewers. Any product that may be evaluated in this article, or claim that may be made by its manufacturer, is not guaranteed or endorsed by the publisher.
