# Corrigendum: Validation of the clinical assessment scale in autoimmune encephalitis in Chinese patients

**DOI:** 10.3389/fimmu.2025.1596678

**Published:** 2025-04-10

**Authors:** Yingchi Zhang, Ewen Tu, Chenxiao Yao, Jia Liu, Qiang Lei, Wei Lu

**Affiliations:** ^1^ Department of Neurology, The Second Xiangya Hospital, Central South University, Changsha, China; ^2^ Department of Neurology, The Second Hospital of Hunan Province, Hunan University of Chinese Medicine, Changsha, China

**Keywords:** autoimmune encephalitis, CASE scores, mRS scores, validation, prognosis

In the published article, there was an error in the legend for

**Table 1** as published. We did not include the copyright information in the legend of **Table 1**. The corrected legend appears below.

**Table 1:** Clinical assessment scale for autoimmune encephalitis (CASE).

Key symptom	Scale	Score
Seizure Memory dysfunction Psychiatric symptoms Consciousness Language problem Dyskinesia/dystonia Gait instability and ataxia Brainstem dysfunction (number of symptoms) Weakness (the mean motor power of all limbs, rounded off)	None Controlled seizures Intractable seizures* Status epilepticus None Mild (does not affect daily activities) Moderate (interferes with daily activities) Severe (no recent memory or unable to communicate) None Mild (no need for medical intervention because it does not affect daily activities) Moderate (need for medical intervention because it interferes with daily activities) Severe (needs continuous care or admission because of psychiatric symptom) or unable to check Alert (opens eyes spontaneously) Drowsy (opens eyes to voice) Stupor (opens eyes to pain) Comatose (does not open eyes) None Mild (slow but able to express sentences) Moderate (unable to express full sentences) Severe (unable to communicate) None Mild dyskinesia (does not affect daily activities) Moderate dyskinesia (interferes with daily activities) Severe dyskinesia causing secondary medical problems Normal Mild, able to walk unassisted Moderate, assisted walking Severe, unable to walk None Gaze paresis Tube feeding Ventilator care due to central hypoventilation Normal (Grade V) Mild (Grade IV) Moderate (Grade III) Severe (≤ Grade II)	0 1 2 3 0 1 2 3 0 1 2 3 0 1 2 3 0 1 2 3 0 1 2 3 0 1 2 3 0 1 2 3 0 1 2 3

The copyright for the CASE score is owned by Seoul National University Hospital. For inquiries, please contact ip@snuh.org or staelee@snu.ac.kr.

**“**Clinical assessment scale for autoimmune encephalitis (CASE). The copyright for the CASE score is owned by Seoul National University Hospital. For inquiries, please contact ip@snuh.org or staelee@snu.ac.kr.”

The authors apologize for this error and state that this does not change the scientific conclusions of the article in any way. The original article has been updated.

